# Exploring patient experiences of surveillance for pancreatic cystic neoplasms: a qualitative study

**DOI:** 10.1136/bmjgast-2023-001264

**Published:** 2024-07-05

**Authors:** Ruth Reeve, Claire Foster, Lucy Brindle

**Affiliations:** 1 University of Southampton, Southampton, UK; 2 Portsmouth Hospitals University NHS Trust, Portsmouth, UK

**Keywords:** PANCREAS, SURVEILLANCE, PRE-MALIGNANCY - GI TRACT

## Abstract

**Background:**

Pancreatic cystic neoplasms (PCN) are considered premalignant conditions to pancreatic adenocarcinoma with varying degrees of cancerous potential. Management for individuals who do not require surgical treatment involves surveillance to assess for cancerous progression. Little is known about patients’ experience and the impact of living with surveillance for these lesions.

**Aims:**

To explore the experiences of patients living with surveillance for PCNs.

**Methods:**

Semi-structured qualitative interviews were conducted with patients under surveillance for pancreatic cystic neoplasms in the UK. Age, gender, time from surveillance and surveillance method were used to purposively sample the patient group. Data were analysed using reflexive thematic analysis.

**Results:**

A PCN diagnosis is incidental and unexpected and for some, the beginning of a disruptive experience. How patients make sense of their PCN diagnosis is influenced by their existing understanding of pancreatic cancer, explanations from clinicians and the presence of coexisting health concerns. A lack of understanding of the diagnosis and its meaning for their future led to an overarching theme of uncertainty for the PCN population. Surveillance for PCN could be seen as a reminder of fears of PCN and cancer, or as an opportunity for reassurance.

**Conclusions:**

Currently, individuals living with surveillance for PCNs experience uncertainty with a lack of support in making sense of a prognostically uncertain diagnosis with no immediate treatment. More research is needed to identify the needs of this population to make improvements to patient care and reduce negative experiences.

**WHAT IS ALREADY KNOWN ON THIS TOPIC**

- The reported incidence of pancreatic cystic neoplasms (PCN) diagnosis has increased due to an increase in medical imaging.
- Current evidence on patient experience for PCN surveillance varies with conflicting reports of levels of cancer worries.
- To date, there have been no qualitative research studies that have examined the patient experiences of PCN surveillance.

**WHAT THIS STUDY ADDS**

- Findings highlight how currently the PCN population experience uncertainty related to a lack of understanding of the diagnosis and potential implications.
- How patients make sense of the PCN diagnosis is influenced by their existing understanding of pancreatic cancer, explanations from clinicians and the presence of coexisting health concerns.
- Uncertainty of living with PCN surveillance may be appraised as a threat to a patient’s life or an opportunity for reassurance.

**HOW THIS STUDY MIGHT AFFECT RESEARCH, PRACTICE OR POLICY**

- Communication of a PCN diagnosis and surveillance management is influential in how patients manage the uncertainty of this diagnosis. Our findings highlight the importance of clear explanations and can help to inform clinicians about how patients respond to a diagnosis of PCN.
- The patient pathway from PCN diagnosis to surveillance is currently complex and inconsistent, highlighting a need for further guidance regarding the optimal management of patients with PCN.

## Introduction

Pancreatic cystic neoplasms (PCN) such as mucinous and non-mucinous cystic neoplasms, are considered premalignant lesions. PCNs are mostly identified incidentally on cross-sectional imaging,^1^ infrequently causing any symptoms prior to diagnosis.^2^ The incidence of PCNs is increasing with the extensive use of cross-sectional imaging, reportedly identified in 20–44% of all abdominal MRI,^2^ where it is now suggested that PCNs can be seen in up to 45% of the general population.^3–6^

The risk of malignancy for PCNs varies depending on PCN characteristics,^7^ with an overall low rate of malignant transformation.^8^ Approximately 8% of pancreatic cancers arise from PCNs.^9^ Given the current prevalence of pancreatic cancer (10 500 new cases/year in the UK),^10^ this means 840 PCNs per year may progress to a cancer diagnosis in the UK alone. Curative treatment and removal of the risk of pancreatic cancer for PCNs involves undergoing high-risk surgical procedures that carry potentially life-limiting effects. Due to an overall low rate of malignant transformation and risks of surgical intervention, PCNs are managed most frequently with imaging surveillance. There are numerous studies exploring the best surveillance procedures and clinical effectiveness.^9^

Despite the increasing prevalence of PCNs in the general population, there is very little evidence exploring the experiences of individuals following a PCN diagnosis.^11^ Existing studies are limited to survey studies that report contrasting findings of cancer worries ranging from 8% to 95%.^11 12^ Furthermore, conflicting findings regarding the effect of surveillance management on individuals’ emotional and psychological symptoms have been reported. Despite one study’s findings of low psychological burden of PCN surveillance,^13^ more recent evidence demonstrates PCN surveillance increased symptoms such as somatisation, depression, anxiety, paranoid ideation and sleep disorders; with higher stress levels and a reduced perception of physical functioning when compared with those managed with surgical treatment.^14^ This highlights that further exploration into the impact of surveillance as a method of disease management is needed.

Evidence relating to patients’ experiences should be considered when introducing healthcare policies and surveillance management. Overall, the limited evidence suggests that experiences of PCN may be varied with some experiencing high cancer worries and psychological burden that negatively affect patients’ quality of life. To date, no qualitative studies have explored these experiences in greater depth. This paper aims to identify the experiences of individuals under surveillance for PCNs, outlining influences on experience and identifying areas for improving patient understanding of PCN diagnosis and surveillance.

## Methods

### Study design

This prospective, multicentre, qualitative research study was conducted between February 2020 and June 2021. To meet the inclusion criteria, participants needed to have been diagnosed with a PCN that is suitable for surveillance, not undergone PCN surgery or had interactions with the interviewer (RR).

## Sampling and recruitment

Participants were purposively sampled prior to recruitment, to capture key characteristics in the population (age, gender, time from diagnosis, management type). Patients were identified in local multidisciplinary team meetings in the participating National Health Service sites in the South of England. Patients were screened and approached by local and clinical teams. Individuals who expressed an interest in participating were given information sheets and reply slips to send onward to the research teams. The interviewer (RR) then contacted the patient to answer questions and arrange a suitable date and time for an interview. Consent was obtained prior to the interview in written format and audio recorded at the beginning of the interview.

## Data collection

Semi-structured individual interviews were conducted to provide rich data relating to individual experiences of diagnosis and lived experience of surveillance. Single interviews were conducted with each participant, each at different time points from diagnosis, to capture how experiences may change over time. The interview format was initially face-to-face, with telephone and video interviewing being introduced as COVID-19 restrictions were introduced. The sample size was guided by similar studies exploring experiences and principles of data saturation,^15 16^ where it was estimated that approximately 30 interviews were needed to allow enough data to generate findings.

An interview topic guide was developed based on findings from a qualitative literature review on patient experiences of surveillance and refined during the data collection period. Questions focused on experiences of diagnosis, living with PCN, surveillance management and influences on their experience and perception of their health following diagnosis (online supplemental file 1). All interviews were conducted by one researcher (RR), audio-recorded with field and reflexive notes also recorded to aid analysis.

10.1136/bmjgast-2023-001264.supp1 Supplementary data

## Data analysis

Interviews were transcribed verbatim and analysed inductively using reflexive thematic analysis (RTA). RTA analytical procedure using Braun and Clarke’s six phases encouraged a systematic, fluid and recursive approach to coding and theme development of the rich, detailed and complex amounts of data within the participant interviews.^17^ Analysis included familiarisation of interview audio, transcripts and field notes, iterative line-by-line coding, generating themes from groups codes of shared overarching concepts, reviewing themes, naming and defining themes and producing the final report. Data analysis was managed via NVivo qualitative data analysis software (V.12).

## Results

### Sample characteristics

27 individuals participated in the interviews. Participant characteristics are detailed in table 1. Data from one interview was not available for analysis due to equipment failure. Participant ages ranged 51–83 years. Male-to-female ratio of participants was 14 M:13 F. All participants resided in England, and all but one participant was of white ethnicity. Time since diagnosis ranged from 62 days to over 7 years.

**Table 1:** Study sample characteristics

Characteristic		Number (%)	Min	Max	Mean
Age	50–59 60–69 70–79 80–89	3(11) 11 (41) 11 (41) 2(11)	51 years	83 years	69 years
Gender	Male Female	14 (51) 13 (49)			
Ethnicity	White Black	26 (96) 1 (4)			
Recruitment site	Site 1 Site 2	14 (52) 13 (48)			
Time since diagnosis			62 days	87 months/ 7 years	1051 days/ 34.5 months/ 2.8 years
Clinician communicating diagnosis Gastroenterologist General surgeon GP Non-specialist in pancreatic diseases No-one (letters only)		4 4 4 10 4			

GP, general practitioner.

The most common clinician seen at diagnosis was a non-specialist in pancreatic diseases (10/26), (including gynaecologists, cardiologists), followed by gastroenterologists, general surgeons and general practitioners (four each). Surveillance methods varied across and within the recruitment sites.

### Summary of results

**Figure 1:**
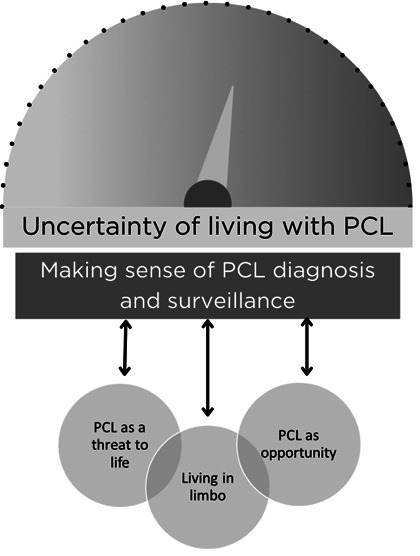
Thematic diagram (uploaded in a separate file). PCL, Pancreatic Cystic Lesion.

One central concept, one main theme and three subthemes were developed from the interviews. The central concept is the overarching recurring pattern within all of the interviews, uncertainty of living with PCN. The main theme from experiences of PCN surveillance was making sense of PCN diagnosis and surveillance. Three subthemes underneath the main theme were: Living in limbo, PCN as an opportunity and PCN as a threat (figure 1). As responses to making sense and living with PCN surveillance went through several stages during individuals’ journeys, it was possible for subthemes to emerge within patient experiences at more than one stage.

#### Central concept: uncertainty of living with PCN

Uncertainty of living with PCN was described by all participants, with levels of uncertainty fluctuating for many in their lives following diagnosis, from fear and panic to very little concern (see online supplemental file 1).

Uncertainty of living with PCN occurred as a result of a lack of knowledge and lack of understanding of the meaning and implications of the diagnosis and the impact that this will have on their lives. Uncertainty is introduced at diagnosis due to the incidental discovery of the lesions; without adequate information uncertainty continued due to a limited understanding of the diagnosis and not knowing what to expect from the disease or surveillance. Individuals with newly diagnosed PCNs have little previous experience of PCNs, therefore make assumptions based on other knowledge or experiences. Uncertainty continued for many as questions remained unanswered, with no guidance on what to expect or what they could be doing to help.

The most prominent causes of uncertainty are:

Uncertainty of the meaning of PCN.

Uncertainty of the purpose of surveillance.

Uncertainty about what to expect.

#### Main theme: making sense of PCN diagnosis and surveillance

A PCN diagnosis was a disruptive life incident for some, resulting in individuals re-evaluating the perception of their health status and attempting to make sense of their situation.

My perception of myself is this really quite strong individual, that nothing was going to knock off her perch until the final days, so having to get to grips with the fact that final days are probably going to be sooner than I’d have liked, came as a bit of a shock. P16

Individuals made sense by contextualising their situation, drawing from and reflecting on their pre-existing knowledge, navigating information provided (or lack of), making comparisons to their experiences for other health concerns and being influenced by explanations from clinicians.

The incidental nature of the PCN diagnosis meant many participants presented initially for investigations concerning other health conditions/indications and had the diagnosis frequently given to them by non-experts of PCN diseases. The method of diagnosis and surveillance method did not influence the experience of living with PCN diagnosis and surveillance. However, the presence of coexisting health concerns was influential to sense-making, individuals contextualised events around the PCN diagnosis and surveillance in relation to their stage of life, wider health and assessed any potential risks involved.

I had enough on my plate, to be honest with you, a couple of years ago with everything else that was going on and that kind of dominated everything […] yeah, maybe it’s something I should worry about but I don’t. P15

Many experiences were shaped by a shared belief that pancreatic cancer causes premature death. A lack of symptoms for pancreatic cancer has often been reported, with the disease being labelled in the public domain as a ‘silent killer’.^18^ Some participants found the knowledge that pancreatic cancer often does not present with symptoms until it is ‘too late’, disturbing.

All I knew of is that (…) pancreatic cancer is a pretty difficult one to deal with and that there’s a less chance of surviving that than on other types. P25

Because of the complicated journey from diagnosis through surveillance with most individuals receiving their diagnosis unexpectedly from other clinical specialties, individuals traversed several events and instances that required sense-making including different clinical departments, clinicians and surveillance tests. Making sense of PCN was therefore an ongoing process that required navigation.

you’re on a rollercoaster aren’t you … you go and get your test done and you don’t until the big man says err that’s alright or [sharp inhale] oh ok or that old sucky lips thing …there’s nothing you can really say …just sitting there nervy really. P1

Explanations and communication between clinicians and patients about the PCN diagnosis and the terminology were influential in how individuals made sense of the uncertainty. The main types of explanations within participant accounts were explanations that normalised the diagnosis through using reassuring descriptions, using terminology that minimised the potential consequences. Such explanations reduced uncertainty and provided reassurance to individuals of the unthreatening nature of PCNs, consequently leading to a positive appraisal of PCN uncertainty.

They just said: “they’re too small to even bother with”. It’s below their limit of needing intervention. P25

Some explanations placed emphasis on the more concerning aspects of the diagnosis and their potential outcome, using negative terminology and phrases. Worrying explanations led to increased fear and concerns that led to the negative appraisal of PCN uncertainty.

He was quite blunt to say the least […] you know he said about uh … it can develop blah blah blah … the operation was quite a nasty operation if you have to have it cut out … you know it was 2 weeks in intensive care … could be over 2 years recovery … he said uh and that’s better than you know if you can’t fix it with cutting it out … but that depends on what size it was […] I can’t remember if it was 30mm or above I would have given you 6 weeks to live. P1

Explanations varied dependant on who participants spoke to. As highlighted in table 1, most participants received their diagnosis from a non-specialist, who were less likely to provide accurate explanations and use more worrying language. Whereas those who saw gastroenterologists and pancreatic surgeons (the most frequent clinician to interact with PCN patients following diagnosis), reported more detailed and reassuring explanations about potential outcomes and consequences of PCNs than those of other clinicians.

Having him explain to me that these things can take quite a long time, I mean you know I was [xx] when it was diagnosed, so I thought well gosh that gets to 77, not so bad, you know, it’s kind of filled me with hope. P16

How individuals make sense of and respond to a PCN diagnosis and surveillance is variable, and may change during an individual’s lifetime, from feeling lucky and reassured, to fearing their premature death and making significant life changes in preparation.

#### Subtheme: living in limbo

Participants described a liminal phase which they themselves called ‘living in limbo’. Uncertainty in the meaning of a PCN was described by participants feeling like they were left hovering in the air. A proportion of the population found themselves in a ‘limbo’, feeling ‘kept in the dark’, being unable to make sense of the meaning or what was happening, resulting in a liminal state of passage.

Living with surveillance, without treatment was confusing for some who were provided verbal information about the potential nature of the PCN diagnosis. Some felt relieved that the diagnosis did not require urgent treatment but felt conflicted by having to live with a diagnosis that had the potential to cause harm in the future.

You’re in turmoil. You--- you’re glad, you don’t want the op but then by the same thing you think: ‘I’ve got this thing inside me and, I want it out.’, you know. So, it’s a confusing feeling. P12

#### Subtheme: PCN as an opportunity

Despite many having fears about their PCN and the potential of cancer, others had a positive outlook on this diagnosis and surveillance. This response was seen when individuals navigated the uncertainty of the circumstances and ambiguous prognosis of their PCN diagnosis, coping with and accepting this phenomenon as an opportunity.

, I’m very thankful that I’m being monitored because if this hadn’t happened when it did in 2013, I would not even to this day know that I’ve got cysts on my pancreas and the ultimate of that is possibly pancreatic cancer discovered too late whereas, I know mine’s going to be discovered if I have it. P22

Interactions between individuals and their clinicians were an important influence for positive appraisals of surveillance. Clinician attitude, terminology and information provided during interactions were influential to individuals’ appraisals of their circumstances. When reassured by clinicians’ attitudes, participants shared a more optimistic outlook, believing that clinicians would do what is best for them, basing their beliefs on previous experiences and interactions.

I mean, you trust these people are doing this every day. They know all of it and you don’t have an inkling of what these things are and what everything involves so, you have to put your trust in them and, you know, I’m sure they act with the best--- your best interests. P12

The relief that their diagnosis was not cancer and considered something with just cancerous potential led to individuals viewing surveillance as an opportunity to detect cancer early or to provide reassurance of their health, which without the initial diagnosis they would not have the opportunity for.

#### Subtheme: PCN as a threat

The seriousness of a PCN diagnosis was contextualised in terms of previous health experiences, comorbidities and interactions with health care professionals (HCPs). The diagnosis with its perceived association with cancer and its potential to cause harm was the main source of concern. As a result, the diagnosis led some to have major concerns for their life and future, with individuals describing these events leading to significant changes in the outlook of their future life.

I mean, when you first get told something like that, you do have that sort of, you know, waking up terrified at night. P23

The power of a potential cancer label becomes evident in the participants’ narratives, especially when the tumour is not removed. For some, it is difficult to live in a situation where the illness is just kept in check, or to be in a position waiting for something to happen. Those who made sense of the PCN diagnosis as a threat were more likely to make lifestyle changes motivated by fear, planning for their demise and preparing for the worst where surveillance acted as a reminder of the potential severity of the threat.

I really felt as though I had to get my life in order. I actually chose to immediately put the house on the market. Decided to move down near my family, near my children, and retire because, I was still working. So, all those things took priority. P10

## Discussion

There is little published research evaluating the levels of distress and anxiety of living with a PCN. Existing studies that have objectively measured potential negative outcomes have not considered the intricacies influencing an individual’s experience, or the causes of distress/anxiety identified relating to PCN diagnosis and surveillance.^19^ Using qualitative interviewing, these findings bring new insights that have previously been unappreciated. The overarching theme was uncertainty in living with the diagnosis and surveillance of a PCN. Within the interviews, individuals living with a PCN were seen to experience uncertainty caused by a lack of understanding of the meaning and significance of a PCN in their lives. In making sense of the diagnosis and surveillance management individuals drew interpretations and understandings from explanations by clinicians, self-sought information on the internet or understandings from personal knowledge and previous experiences. As a result, making sense of PCN uncertainty was an ongoing complex process that varied between participants, depending on how an individual appraises their diagnosis.

The qualitative findings from individuals who were fearful of the PCN diagnosis and surveillance, bring nuanced insights into the existing evidence of the PCN experience, expanding on the how and what is frightening for those living with this diagnosis. In Shieh’s research, their findings demonstrated that a proportion of individuals living with a PCN diagnosis can be distressed, with many having a heightened perception of cancer risk associated with their diagnosis.^20^ Where our qualitative findings illustrate in more detail how and why these responses and interpretations arise, highlighting how individuals often rely on their own knowledge to make sense of their circumstance and draw on the explanations from clinicians to assess their risk.

Individuals with a PCN diagnosis attempted to make sense of their circumstances by contextualising their situation. With a limited understanding of PCNs prior to diagnosis, many participants feared the PCN diagnosis was associated with pancreatic cancer, whether this was explicitly explained to them or not. Previous knowledge or experience of pancreatic cancer was an influential factor in individuals’ perception of danger from their diagnosis, with some reporting living in fear for some time after first learning about their diagnosis. In a study of PCN patients evaluating reasons for choosing surgery following diagnosis in the USA, fear of cancer was identified as an important factor.^19^ What has not been considered in previous studies, however, is the impact of fearing cancer while living with a PCN when surgery is not a treatment option offered, particularly when patients have first-hand experiences and/or some knowledge of pancreatic cancer.

Knowledge and understanding of the implications of a PCN diagnosis was minimal compared with the awareness of pancreatic cancer that participants had within the study population, resulting in individuals associating a PCN diagnosis with pancreatic cancer. Pancreatic cancer is negatively represented in the media where it is associated with poor prognosis and premature death, therefore receiving a diagnosis of a PCN had the potential to be a biographically disruptive event. The emotional consequences of living with a PCN diagnosis that may develop into a cancer has not been previously considered in research within this population. The findings from this study highlight the need for more work within clinical environments to ensure that patients receiving a diagnosis of a PCN are supported to understand the PCN diagnosis, and that disruption to their lives is minimised where possible. The risk of developing pancreatic cancer from a PCN is not negligible but not significant enough for all patients to be given the choice of invasive surgery, despite the cancer fears experienced by some people living with PCNs. This study has highlighted the experiences of patients under surveillance for a PCN diagnosis, follow on from this study should identify the specific needs of patients to improve care and reduce the uncertainty that negatively impacts patients life.

Clinicians’ explanations were influential, however, currently inconsistent, with contrasting explanations resulting in varied responses (such as fear). Patients’ sense-making was influenced by clinicians’ level of concern, because of the trust that individuals also placed in their clinicians. Individuals trusted that clinicians were more knowledgeable than themselves, had their best interests at heart and would provide them with the best care that was available. Differences in explanations may be due to the varied level of knowledge or expertise of PCNs among the clinicians, with most participants receiving their diagnosis from a non-pancreas specialist. The clinician’s own personal perspective of a PCN diagnosis and its associated risk could have influenced how they communicate the diagnosis to their patient. Overall, similar to the wider evidence in the general and premalignant populations, certain terms such as ‘lump’, ‘growth’ and ‘mass’, were negatively associated with cancer,^21 22^ whereas terms such as ‘cyst’ had fewer negative associations. Our findings highlight research on clinician communication education or interventions and patient information and implementation of patient information is required to identify their influence on appraisal of PCN uncertainty and potentially improve patient experience.

Previous research reviewing PCN surveillance suggests that patients accept surveillance, without exploring what the consequence of surveillance is for patients.^13^ Our findings present the experiences of those who appraise PCN uncertainty as an opportunity, seeking reassurance from monitoring, for the first time providing explanations for the findings from previous research which found that patients were happy to undertake surveillance with low degrees of cancer worry.^13^ Taking a qualitative and subtle realist approach has allowed further exploration of the existing quantitative findings and identified key features of the experience of PCN diagnosis and surveillance such as the presence of coexisting health concerns and the different ways individuals made sense of PCN uncertainty.

A PCN diagnosis is made incidentally in almost all cases; this means that all individuals are having medical investigations or tests for other clinical reasons at the time of PCN diagnosis. As a result, almost everyone diagnosed with a PCN is also navigating and/or living with other health conditions. The addition of a PCN diagnosis and surveillance to the lives of people living with other illnesses has not been previously explored. Our qualitative findings of PCN surveillance show the significance of a PCN diagnosis can vary, where individuals contextualise their PCN diagnosis by considering existing health conditions and making judgements on the risk of a PCN diagnosis to their life and well-being.

The impact of a PCN diagnosis has not previously been considered with regards to how it may increase the existing health burden for individuals. Globally, the number of people living with multimorbidity is increasing.^23^ Furthermore, the number of screening tests undertaken with the aim to detect cancers sooner and at earlier stages of the disease process is increasing. As a result, patients are now beginning to live with several disease processes at once, requiring an increasing volume of work and management. The accumulation of workload for multimorbidity can result in patients being expected to manage an increasing number of health-related activities,^23^ where in general, HCPs are trained to delegate work to patients in line with guidelines.^24^ Understanding and appreciating the impact of increasing workload for patients living with multiple health conditions is important for clinicians when developing guidance and health policy for disease management.^25^

Potential limitations of this study are the limited ethnic diversity of the sample. Most of the participants were recruited from one geographical region which was predominantly white British, as such, a limitation of this research is that the experiences of those from non-white groups are not reflected within the study findings. Previous research has demonstrated that individuals from ethnic minority communities experience cultural issues in relation to cancer screening,^26 27^ and have a higher perceived cancer stigma, with more confusion leading to fear of cancer.^28 29^ Therefore, further research, with cohorts of individuals from a wider ethnic background through a multicentre study should be conducted to evaluate the transferability of these findings to the wider population. A strength of this study however is the qualitative methodology, and choice of cross-sectional purposive sampling strategy. Resultingly, experiences were collected from the point of diagnosis to following years of surveillance, and from multiple sites including a general hospital and tertiary centre.

## Conclusion

This study describes the experiences of individuals living with PCN surveillance. Our findings demonstrate that individuals who receive a PCN diagnosis have to navigate a complex journey to surveillance following an incidental diagnosis. For the first time we have demonstrated that receiving a PCN diagnosis can be experienced as a disruptive life event. The disruption is caused by uncertainty relating to the ambiguous nature of disease progression, the lack of understanding of the implications and the meaning of the diagnosis to one’s life. Through the in-depth exploration of the patient experience, we have identified how clinicians influence individuals’ uncertainty, their perception of what the PCN means to their life and their appraisal of potential danger. The findings relating to these experiences underline the importance of suitable and appropriate information provision to aid people’s sense-making and to reduce uncertainty. More work is now needed to identify the information and support needs of the PCN population to make recommendations that can improve PCN surveillance experiences.

## Data Availability

Data are available upon reasonable request. All data relevant to the study are included in the article or uploaded as supplementary information. Extracts of data can be found in the supplementary information.
