# Supplementary material for: Exploring patient experiences of surveillance for pancreatic cystic neoplasms: a qualitative study
Source: BMJ Open Gastroenterol. 2024 Jul 5;11(1):e001264. doi: 10.1136/bmjgast-2023-001264 (PMC11227750; doi:10.1136/bmjgast-2023-001264)
Supplement: Supplementary data [file bmjgast-2023-001264supp001.pdf]

Supplemental information

Participant quotes

Theme		Sample Quotes
Uncertainty of living with PCN	Causes of uncertainty:<br>- Uncertainty of the meaning of PCN.	*"That's all I've been told, have another scan and it'll go you know, didn't even say, well I said to her you know, erm, what does it mean, she said well until we get the scan done, we don't know." P18*
	Causes of uncertainty:<br>- Uncertainty of the purpose of surveillance.	*"Why are they actually doing it I mean what is the purpose…so yeh they could explain it a little bit more." P6*
	Causes of uncertainty:<br>- Uncertainty about what to expect.	*"Well, I just really want to know going forward what to expect. That--- that's my biggest question…So, how it might affect me going forward really is what I'm most concerned about." P24*
Making sense of PCN diagnosis and surveillance	Co-existing health concerns was influential to sense making.	*"I had enough on my plate, to be honest with you, a couple of years ago with everything else that was going on and that kind of dominated everything […] yeah, maybe it's something I should worry about but I don't." P15*
	Knowledge of pancreatic cancer.	*"All I knew of is that (…) pancreatic cancer is a pretty difficult one to deal with and that there's a less chance of surviving that than on other types." P25*<br><br>*"I mean I obviously I'm aware of the symptoms, although by the time you have symptoms it's a late in the day" P16*
	Explanations influenced appraisal of PCN diagnosis.	*"They just said: "they're too small to even bother with". It's below their limit of needing intervention." P25*<br><br>*"He was quite blunt to say the least […] you know he said about uh … it can develop blah blah blah … the operation was quite a nasty operation if you have to have it cut out … you know it was 2 weeks in intensive care … could be over 2 years recovery … he said uh and that's better than you know if you can't fix it with cutting it out … but that depends on what size it was […] I can't remember if it was 30mm or above I would have given you 6 weeks to live." P1*
Sub theme: Living in limbo	Conflicted feelings having to live with a diagnosis carrying potential to cause harm.	*"you're panicking and you think: 'But, I've got this lump inside me.', you know, and you're--- you're so keyed up because it was a big thing for me to think I had to go through this op, you know. It was really weighing on me […] I'm thinking: 'Oh, but I've got this lump here.*

		Suppose something happens?', you know. So, you're--- you're in turmoil. You--- you're glad, you don't want the op but then by the same thing you think: 'I've got this thing inside me and, I want it out.', you know. So, it's a confusing feeling. Very confusing." P12
Sub theme: PCN as an opportunity	Accepting a PCN diagnosis and surveillance as an opportunity for reassurance.	*"If anything is found then something can be done about it. I mean, I'm very thankful that I'm being monitored because if this hadn't happened when it did in 2013, I would not even to this day know that I've got cysts on my pancreas and the ultimate of that is possibly pancreatic cancer discovered too late whereas, I know mine's going to be discovered if I have it." P22*
Sub theme: PCN as a threat	PCN diagnosis caused major concerns for their future, resulting in changes within their lifestyle.	"*I've probably been more focused on planning for the non-future than I might have been […] Like recently trying to setup a small trust fund for my grandchildren, things like that, because I think rightly, I'm assumed rightly, I'm not going to be here when they're older […]That's probably the only change, am I slightly less optimistic than I used to be." P16*<br><br>*"So I don't look forward to any kind of medical test to be truthful, but this one also carries the weight of well something may have changed. The cysts could have enlarged, outlook could be grim" P16*

Supplemental information

Interview topic guide

Introduction and opening remarks

- Introduce self and tell participant you will begin recording
- Achieve informed consent verbally, take participant through information sheet and consent form, ensuring participant is aware of the broad aims of the study, and their rights as research participants. Fill in consent form if face to face (if telephone/video – check details on copy received).
- Explain the value of the voice and experience of the participant to the aims of the study.
- Ensure the participant is comfortable to begin the interview and give them the opportunity to ask any questions before beginning.

Topic 1. Diagnosis

Can you tell me how you first found out you had a cyst in your pancreas?

Prompt – when, who, what did they say

What were your feelings when you heard?

Prompt – why do you think you felt like that? what are your concerns? Why does that concern you?

Can you describe in your own words what a cyst is?

Topic 2. Feelings & Impact of illness

What is your understanding of the purpose of surveillance?

Probe – what is your understanding of what they are looking for? What is your understanding about any risks that are being monitored?

What are your feelings about having surveillance?

Prompt – How would you feel if they did not monitor? Have you considered what you would like to happen in an ideal world?

Did you discuss the diagnosis or your feelings with anybody else?

Prompt – who, what was their reaction?

How do you feel when a surveillance event comes up?

Prompt – why do you think you feel like that?

What are your feelings when during the surveillance test?

Prompt – why do you think you feel like that? How do you feel during this compared to other tests that you have? What are you told at the test? Is there anything that you would change if you could?

Can you describe how you feel while waiting for the results?

Prompt – How do you feel after the test? How do you find out the results? Why do you think you feel like that? How do you feel after you find out the results?

Topic 3. Feelings towards the needs & support

How have you been communicated with?

Prompt – how were you first told? How are you told results since? What are your feelings about how you are told? What is your preference?

What information have you received about the diagnosis?

Prompt – How were you given information? What types of information? What is your preference for information?

What support have you had during surveillance?

Prompt – how does that make you feel? What support would you have liked?

In an ideal scenario what would you change to have given you a better experience?

Prompt – what time would have been best to receive that?

Topic 4. Past, present & future

On a day to day basis how do you feel about/deal with the diagnosis?

Prompt – do you have any particular strategies for helping? Or coping?

Have your feelings or concerns changed over time?

Prompt – how do you think they have changed? Why do you think they have changed?

Has the diagnosis made a difference to how you see yourself?

Prompt – In what way would you say it is different now? How would you say that it has changed?

How do you view your own health now?

Prompt - Do you see yourself as being ill?

How do you feel about the future?

Prompt – how do you see this playing out? How does that make you feel?

End of interview and closing remarks

- Everything discussed today is completely confidential and will be untraceable to you. Having said that is there anything we discussed that you would like to withdraw?
- Was there anything you would like to seek clarification on?
- What is the best way to contact you going forward?
- Thank you for your time today.
